# A nomogram based on serum cystatin C for predicting acute kidney injury in patients with traumatic brain injury

**DOI:** 10.1080/0886022X.2021.1871919

**Published:** 2021-01-21

**Authors:** Ruo Ran Wang, Min He, Xiying Gui, Yan Kang

**Affiliations:** a Department of Critical Care Medicine, West China Hospital, Sichuan University, Chengdu, China; b Department of Critical Care Medicine, Tibet Autonomous Region People’s Hospital, Lhasa, China

**Keywords:** Cystatin C, acute kidney injury, traumatic brain injury, biomarker

## Abstract

**Background:**

Acute kidney injury (AKI) is a common complication in traumatic brain injury (TBI) patients and is associated with unfavorable outcome of these patients. We designed this study to explore the value of serum cystatin C, an indicator of renal function, on predicting AKI after suffering TBI.

**Methods:**

Patients confirmed with TBI and hospitalized in the West China Hospital of Sichuan University between January 2015 and December 2019 were included. Patients were divided into two groups according to occurrence of AKI. Univariate and multivariate logistic regression analyses were sequentially utilized to find risk factors of AKI in included TBI patients. Nomogram composed of discovered risk factors for predicting AKI was constructed. Receiver operating characteristics (ROC) curves were drawn and area under the ROC curve (AUC) were calculated to evaluate the predictive value of cystatin C alone and the constructed nomogram.

**Results:**

Among 234 included TBI patients, 55 were divided into AKI group. AKI group had shorter length of stay (*p* < 0.001) and higher in-hospital mortality (*p* < 0.001). Multivariate logistic regression analysis showed absolute lymphocyte count (*p* = 0.034), serum creatinine (*p* < 0.001), serum cystatin C (*p* = 0.017) and transfusion of red blood cell (*p* = 0.005) were independently associated with development of AKI after TBI. While hypertonic saline use was not associated with the development of AKI (*p* = 0.067). The AUC of single cystatin C and predictive nomogram were 0.804 and 0.925, respectively.

**Conclusion:**

Higher serum cystatin C is associated with development of AKI in TBI patients. Predictive nomogram incorporating cystatin C is beneficial for physicians to evaluate possibilities of AKI and consequently adjust treatment strategies to avoid occurrence of AKI.

## 1. Introduction

As a widely concerned public health problem, traumatic brain injury (TBI) lead to terrible mortality and disability of victims. It is estimated that approximately sixty-nine million people would suffer TBI each year all over the world [1]. The high mortality of TBI patients not only be attributable to the initial severity of brain injury, but also systemic complications secondary to brain injury [2,3]. Acute kidney injury (AKI) is one form of these systemic complications manifested as rapid occurrence of renal dysfunction. AKI after TBI has been reported developing in 7.6% to 23% patients and is correlated with mortality, functional outcome and length of hospital stay in TBI patients [4–7]. Mechanisms involved in development of AKI after TBI are diversified, which included systemic inflammation response, neuroendocrine hormone release, hypoperfusion and iatrogenic factors such as blood transfusion, drugs reducing intracranial pressure and usage of nephrotoxic antibiotics [8–11]. In view of the unfavorable outcome caused by AKI, exploring novel and available biomarkers to predict the possible occurrence of AKI in early stage and consequently avoid medical treatments adverse to normal renal function is beneficial for outcome and recovery of TBI patients.

The cystatin C, a kind of endogenous cysteine proteinase inhibitor taking part in the catabolism of intracellular proteins and peptides, has been demonstrated as an effective marker of renal function [12,13]. Compared with creatinine, life cycle of serum cystatin C is shorter which makes it capable of detecting declined renal function earlier than serum creatinine and more applicable in emergency condition [13–15]. In addition, cystatin C is less likely to be influenced by muscle wasting, malnutrition, age and sex than creatinine, which indicates it may be more reliable and accurate in reflecting glomerular filtration rate (GFR) [12,16–18]. Predictive value of serum cystatin C on evaluating occurrence of AKI has been confirmed in various groups of patients including critically ill patients, patients undergoing cardiac surgery and those receiving radiocontrast medium [19–25]. However, there are no studies exploring the association between serum cystatin C level and occurrence of AKI after TBI. Therefore, we designed this study to testify the hypothesis that serum cystatin C is an effective marker of AKI in TBI patients.

## 2. Materials and methods

### 2.1. Patients

This observational study was retrospectively conducted in West China hospital of Sichuan University. We included TBI patients treated in neuro-intensive care unit (NICU) of West China hospital between January 2015 and December 2019. TBI patients hospitalized in our NICU were mostly confirmed with moderate to severe TBI patients (GCS <13) who required continuous intensive care and organ function support. Diagnoses of included patients were confirmed by analyzing radiological signs in computed tomography (CT) scans. Patients were excluded from this study if they met following criteria: (1) admitted to West China hospital 4 h after suffering injury; (2) hospitalized in West China hospital less than 48 h; (3) history of severe underlying diseases such as severe cardiovascular diseases, stroke, malignant hypertension, severe metabolic diseases, severe hepatorenal diseases and active cancer; (4) incomplete clinical data included in this study; (5) history of surgical operation within 4 weeks before TBI. Finally, 234 patients were included in this single center retrospective study. Our study was approved by the ethics committee of West China hospital (2018-359) and conducted accorded with Declaration of Helsinki. Informed consent form about clinical study of each patients was routinely signed by patients themselves or their legally authorized representatives during their hospitalization.

### 2.2. Data collection

All clinical and laboratory variables included in this study were collected from records of electronic medical record (EMR) system in West China hospital. Demographic variables such as age and gender, injury mechanisms, vital signs on admission including systolic blood pressure, diastolic blood pressure, heart rate, body temperature and respiratory rate were collected in this study. Occurrence of anisocoria and Glasgow Coma Scale (GCS) on admission were also recorded. Variables of blood routine and blood biochemistry examination including serum cystatin C level were obtained by analyzing blood samples collected at admission. Serum concentrations of cystatin C was detected using the Cobas 8000 chemistry autoanalyzer (Roche Diagnostics, Switzerland). Anatomic injury types including epidural hematoma, subdural hematoma, intracerebral hematoma, subarachnoid hemorrhage, intraventricular hemorrhage and diffused axonal injury were recorded according to findings of CT scans. Red blood cell (RBC) transfusion, usage of drugs for reducing intracranial pressure and types of surgical operation were included. Outcome of this study was the occurrence of AKI in TBI patients during their hospitalization. AKI was diagnosed based on the KIDGO criteria from the second day after admission to discharge.

### 2.3. Statistical analysis

We used Kolmogorov-Smirnov test to testify the normality of included variables. Normally distributed variables and non-normally distributed variables were presented in the form of mean ± standard deviation and median (interquartile range), respectively. Categorical variables were shown as counts (percentage). Student’s t-test was utilized to compare the difference between two groups of normally distributed variables. And Mann-Whitney U test was performed to analyzed the difference between two groups of non-normally distributed variables. Chi-square test or Fisher exact test were conducted to confirm the difference between two groups of categorical variables. Univariate and multivariate logistic regression analysis were sequentially performed to explore independent risk factor for AKI after TBI. Odds ratio (OR) and 95% confidence intervals (CI) of each risk factors were calculated and presented. Discovered independent risk factors were combined to construct logistic regression model for predicting AKI after TBI. For convenient clinical use, the predictive model was visualized using nomogram. Receiver operating characteristic (ROC) curves of single and constructed predictive model were drawn and area under the ROC curve (AUC) value of them were calculated. Z test was conducted to verify the difference of AUC between single serum cystatin C and the predictive model.

Two-tailed P value <0.05 was considered to be statistically significant. SPSS 22.0 (IBM Corp., Armonk, NY, USA) and R (version 3.6.1; R Foundation) was used to carry out all statistical analyses and figures drawing.

## 3. Results

### 3.1. Comparison of baseline characteristics between non-AKI and AKI patients

A total of 234 patients confirmed with TBI were included in this study. Patients were divided into two groups based on whether they developed AKI during hospitalization. As a result, 179 patients were included in non-AKI group and 55 patients were selected into AKI group with AKI incidence of 23.5% (Table 1). The average age of overall patients was 44 and the male ratio of overall was 77.8%. Among recorded injury mechanisms, motor vehicle collision was the leading cause of initial brain injury. High falling and stumbling injury ranked the second and third, respectively. Compared with non-AKI group, AKI groups showed no significant difference on admission vital signs including systolic blood pressure, diastolic blood pressure, heart rate, body temperature and respiratory rate. Occurrence rate of anisocoria also did not significantly differ between non-AKI group and AKI group (35.8% vs 36.4%, *p* = 0.934). While AKI group had significantly lower GCS score than non-AKI group (5 vs 6, *p* < 0.001). Results of routine laboratory tests presented that AKI group had lower platelet count (89 vs 125, *p* < 0.001), hemoglobin (90 vs 92, *p* = 0.001), fibrinogen (2.22 vs 3.07, *p* = 0.044), albumin (29.2 vs 31,8, *p* = 0.004) and cholesterol (2.02 vs 2.82, *p* < 0.001) than non-AKI group with statistical significance. However, the level of lactate dehydrogenase (LDH) (474 vs 363, *p* < 0.001), blood urea nitrogen (BUN) (9.34 vs 5.77, *p* < 0.001), serum creatinine (SCr) (124 vs 65, *p* < 0.001) and serum cystatin C (1.09 vs 0.72, *p* < 0.001) were obviously higher in AKI group than non-AKI group. Radiological findings showed that non-AKI group was more frequently observed with epidural hematoma (16.2% vs 1.8%, *p* = 0.001). Other types of brain injury did not significantly differ between these two groups. Records of treatments indicated that patients with AKI were more likely to receive red blood cell (RBC) transfusion (52.7% vs 31.8%, *p* = 0.006) and less likely to be treated with hypertonic saline (18.2% vs 41.3%, *p* = 0.376). Compared with non-AKI group, AKI group had shorter length of hospital stay (9 vs 24, *p* < 0.001) and higher mortality rate (74.5% vs 30.2%, *p* < 0.001).

**Table 1.** Baseline characteristics of non-AKI group and AKI group in included TBI patients.

Variables	Overall patients (*N* = 234)	Non-AKI group (*n* = 179)	AKI group (*n* = 55)	*p* Value
Age (years)	44 (28–59)	43 (27–56)	47 (34–64)	0.092
Sex (male ratio)	182 (77.8%)	136 (76%)	46 (83.6%)	0.220
Injury mechanism				
Traffic accident	148 (63.2%)	112 (62.6%)	36 (65.5%)	0.697
High falling	49 (20.9%)	37 (20.7%)	12 (21.8%)	0.855
Stumble	22 (9.4%)	15 (8.4%)	7 (12.7%)	0.349
Others	15 (6.4%)	15 (8.4%)	0	0.025
Vital signs on admission				
Systolic blood pressure (mmHg)	123 (108–141)	125 (110–141)	120 (103–146)	0.326
Diastolic blood pressure (mmHg)	73.79 ± 16.58	74.07 ± 15.32	72.87 ± 20.29	0.687
Heart rate (min^−1^)	98 (81–120)	96 (80–116)	107 (86–121)	0.066
Body temperature (°C)	36.8 (36.5–37.1)	36.8 (36.5–37.1)	36.8 (36.5–37.8)	0.610
Respiratory rate (min^−1^)	20 (17–23)	20 (17–23)	20 (16–22)	0.615
Anisocoria	84 (35.9%)	64 (35.8%)	20 (36.4%)	0.934
GCS	6 (4–8)	6 (5–9)	5 (3–6)	<0.001
Laboratory tests				
White blood cell (10^9^/L)	15.09 (11.14–20.43)	14.84 (11.27–19.49)	15.76 (10.58–23)	0.362
Neutrophil (10^9^/L)	11.61 (8.64–15.77)	11.38 (8.77–15.60)	12.32 (7.67–17.54)	0.927
Lymphocyte (10^9^/L)	0.82 (0.54–1.16)	0.84 (0.55–1.22)	0.75 (0.46–1.02)	0.071
Platelet (10^9^/L)	115 (78–169)	125 (86–179)	89 (54–136)	<0.001
Hemoglobin (g/L)	88 (77–106)	92 (79–110)	80 (72–91)	0.001
Fibrinogen (mg/L)	2.88 (1.68–4.27)	3.07 (1.78–4.45)	2.22 (1.23–4.06)	0.044
Albumin (g/L)	31.2 ± 5.9	31.8 ± 6.0	29.2 ± 5.4	0.004
Cholesterol (mmol/L)	2.66 (1.95–3.51)	2.82 (2.21–3.67)	2.02 (1.44–2.76)	<0.001
Lactate dehydrogenase (U/L)	380 (291–533)	363 (270–491)	474 (344–726)	<0.001
Blood urea nitrogen (mmol/L)	6.11 (4.70–8.67)	5.77 (4.43–7.77)	9.34 (5.8–14.61)	<0.001
Serum creatinine (umol/L)	71 (56–98)	65 (51–81)	124 (98–172)	<0.001
Serum cystatin C (mg/L)	0.78 (0.63–0.97)	0.72 (0.60–0.89)	1.09 (0.85–1.65)	<0.001
Radiological findings				
Epidural hematoma	30 (12.8%)	29 (16.2%)	1 (1.8%)	0.001
Subdural hematoma	93 (39.7%)	71 (39.7%)	22 (40%)	0.965
Intracerebral hematoma	81 (34.6%)	61 (34.1%)	20 (36.4%)	0.756
Subarachnoid hemorrhage	102 (43.6%)	80 (44.7%)	22 (40%)	0.538
Intraventricular hemorrhage	15 (6.4%)	12 (6.7%)	3 (5.5%)	1.000
Diffused axonal injury	35 (15%)	29 (16.2%)	6 (10.9%)	0.321
Treatment options				
Transfusion of RBC	86 (36.8%)	57 (31.8%)	29 (52.7%)	0.006
Mannitol	174 (74.4%)	136 (76.0%)	38 (69.1%)	0.313
Fructose glycerol	25 (10.7%)	20 (11.2%)	5 (9.1%)	0.657
Hypertonic saline	84 (35.9%)	74 (41.3%)	10 (18.2%)	0.001
Decompressive craniectomy	86 (36.8%)	63 (35.2%)	23 (41.8%)	0.376
Hematoma evacuation	98 (41.9%)	75 (41.9%)	23 (41.8%)	0.991
Length of ICU stay (days)	13 (2–25)	14 (4–25)	5 (2–27)	0.057
Length of hospital stay (days)	22 (8–39)	24 (11–41)	9 (3–29)	<0.001
In-hospital mortality	95 (40.6%)	54 (30.2%)	41 (74.5%)	<0.001

GCS: Glasgow Coma Scale; RBC: red blood cell.

### 3.2. Univariate and multivariate logistic regression analysis of risk factors for AKI

We primarily performed univariate logistic regression and found that GCS (OR = 0.735, *p* < 0.001), absolute lymphocyte count (OR = 0.520, *p* = 0.042), platelet (OR = 0.993, *p* = 0.004), hemoglobin (OR = 0.981, *p* = 0.012), albumin (OR = 0.926, *p* = 0.005), cholesterol (OR = 0.479, *p* < 0.001) and epidural hematoma (OR = 0.096, *p* = 0.023) were negatively correlated with occurrence of AKI in included TBI patients (Table 2). While LDH (OR = 1.002, *p* = 0.001), BUN (OR = 1.334, *p* < 0.001), SCr (OR = 1.061, *p* < 0.001) and serum cystatin C (OR = 51.742, *p* < 0.001) were positively related with development of AKI in included TBI patients. Subsequently, we included significant factors above mentioned in univariate analysis into multivariate logistic regression. After adjusting confounding effects, only lymphocyte count (OR = 0.324, *p* = 0.034), SCr (OR = 1.052, *p* < 0.001), serum cystatin C (OR = 8.801, *p* = 0.017) and RBC transfusion (OR = 4.212, *p* = 0.005) were significantly associated with development of AKI during hospitalization.

**Table 2.** Univariate and multivariate logistic regression analysis of risk factors for AKI after TBI.

	Univariate logistic regression analysis			Multivariate logistic regression analysis		
Variables	OR	95% Cl	p	OR	95% Cl	*p* Value
Age (years)	1.015	0.999–1.032	0.065			
Sex (male)	1.616	0.732–3.569	0.235			
Systolic blood pressure (mmHg)	0.997	0.985–1.009	0.597			
Diastolic blood pressure (mmHg)	0.996	0.978–1.014	0.638			
Heart rate (min^-1^)	1.009	0.998–1.021	0.104			
Body temperature (°C)	1.108	0.804–1.528	0.530			
Respiratory rate (min^-1^)	1.004	0.950–1.061	0.894			
Anisocoria	1.027	0.548–1.925	0.934			
GCS	0.735	0.633–0.854	<0.001			
White blood cell (10^9^/L)	1.030	0.987–1.075	0.177			
Neutrophil (10^9^/L)	0.997	0.977–1.019	0.815			
Lymphocyte (10^9^/L)	0.520	0.277–0.976	0.042	0.324	0.114–0.920	0.034
Platelet (10^9^/L)	0.993	0.988–0.998	0.004			
Hemoglobin (g/L)	0.981	0.966–0.996	0.012			
Fibrinogen (mg/L)	0.877	0.743–1.035	0.120			
Albumin (g/L)	0.926	0.877–0.977	0.005			
Cholesterol (mmol/L)	0.479	0.336–0.684	<0.001			
Lactate dehydrogenase (U/L)	1.002	1.001–1.003	0.001			
Blood urea nitrogen (mmol/L)	1.334	1.199–1.485	<0.001			
Serum creatinine (umol/L)	1.061	1.043–1.079	<0.001	1.052	1.031–1.074	<0.001
Serum cystatin C (mg/L)	51.742	13.176–203.187	<0.001	8.801	1.487–52.087	0.017
Epidural hematoma	0.096	0.013–0.720	0.023			
Subdural hematoma	1.014	0.547–1.879	0.965			
Intracerebral hematoma	1.105	0.589–2.076	0.755			
Subarachnoid hemorrhage	0.825	0.446–1.525	0.540			
Intraventricular hemorrhage	0.803	0.218–2.955	0.741			
Diffused axonal injury	0.633	0.248–1.615	0.339			
Transfusion of RBC	2.387	1.290–4.419	0.006	4.212	1.541–11.515	0.005
Mannitol	0.707	0.363–1.377	0.308			
Fructose glycerol	0.795	0.284–2.227	0.663			
Hypertonic saline	0.315	0.149–0.666	0.002	0.357	0.119–1.074	0.067
Decompressive craniectomy	1.323	0.714–2.454	0.374			
Hematoma evacuation	0.997	0.540–1.839	0.991			

OR: odds ratio; Cl: confidence interval; GCS: Glasgow Coma Scale; RBC: red blood cell.

### 3.3. Value of serum cystatin C and constructed logistic model for predicting AKI in TBI patients

**Figure 1.**
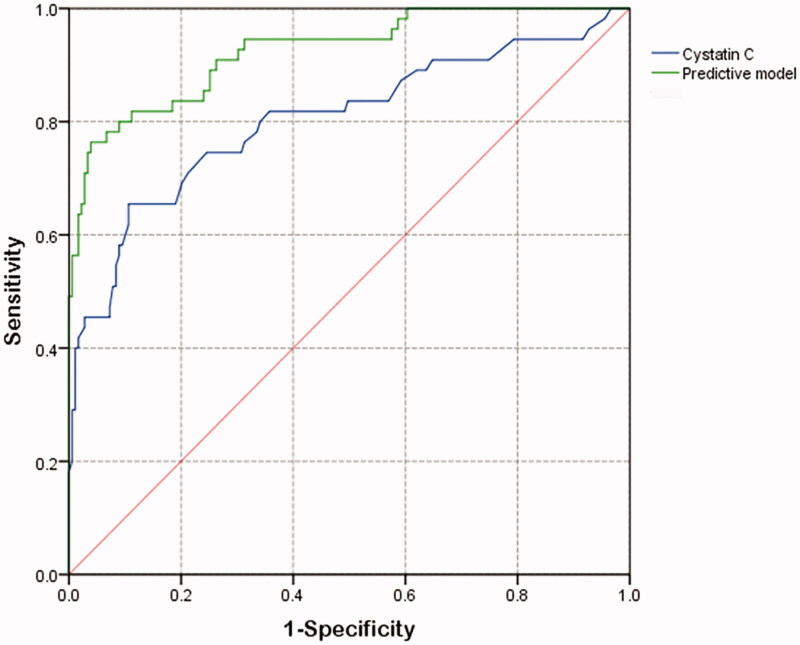
ROC curves of cystatin C and constructed predictive model for predicting AKI after TBI.

**Figure 2.**
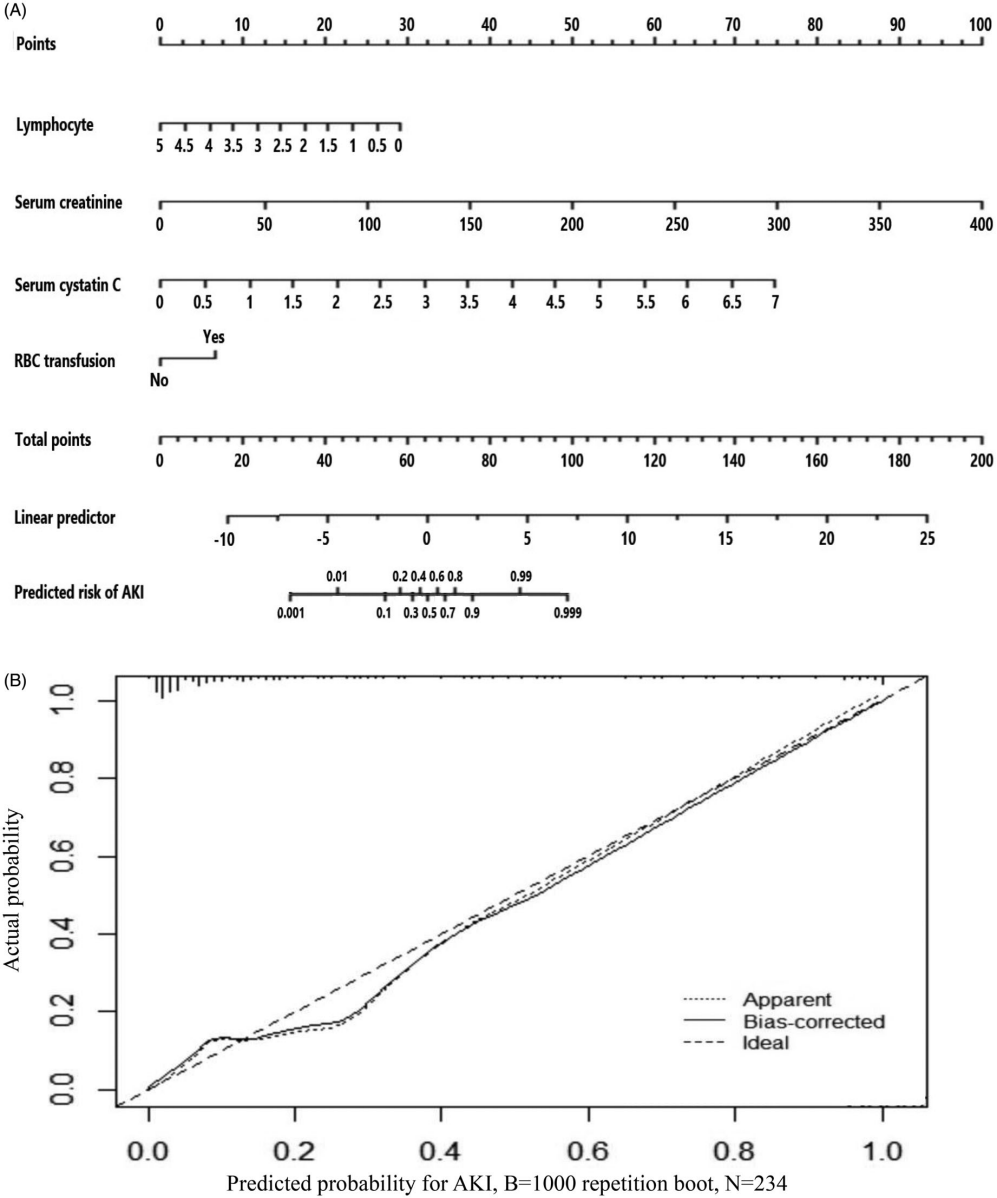
(A) Nomogram of the constructed logistic model for predicting AKI in included TBI patients. The nomogram consisted of four variables including lymphocyte, serum creatinine, serum cystatin C and records of RBC transfusion. Based on the sum of corresponding points of each variable, total points could be calculated and therefore evaluated the risk of AKI. (B) Calibration plot for predicting AKI in included TBI patients.

The AUC of ROC curves (Figure 1) about predicting AKI was listed in Table 3. The AUC of serum cystatin C alone was 0.804, which was lower than 0.925 of logistic predictive model comprised of lymphocyte count, SCr, cystatin C and RBC transfusion. For convenient clinical use, we drawn readily visualized nomogram of this predictive model (Figure 2(A)). The calibration curve of this model showed good consistency between actual probabilities and predicted probabilities of AKI (Figure 2(B)).

**Table 3.** Predictive value of cystatin C and constructed model for predicting AKI after TBI.

	AUC	95% Cl	Sensitivity	Specificity
Cystatin C	0.804	0.727–0.880	0.655	0.894
Predictive model	0.925	0.883–0.967	0.764	0.961

AUC: area under the ROC curve; Cl: confidence interval.

## 4. Discussion

This is the first study to confirm the value of serum cystatin C on predicting AKI in TBI patients. In this study, we found that lymphocyte count, SCr, cystatin C and RBC transfusion were independently associated with development of AKI in TBI patients. The single cystatin C level was effective in predicting AKI while combining four factors above mentioned was more accurate than cystatin C alone in evaluating the possible occurrence of AKI in TBI patients.

The development of AKI is a prevalent non-neurologic complication in TBI patients, which has been reported occurring in 7.6% to 23% TBI patients [4–7]. Multiple mechanisms may be involved in the development of AKI after TBI including massive catecholamine release, various inflammatory mediators induced systemic inflammation [3,26]. The catecholamine surge, mainly characterized as a massive release of norepinephrine, epinephrine, and dopamine is a necessary pathophysiologic process after TBI and could stimulate the activation of renin-angiotensin-aldosterone-system (RAAS) which subsequently aggravates the deteriorating renal function [3,27,28]. In addition, many kinds of inflammatory mediators including IL-1β, IL-6 and CRP would be released from injured brain tissue to circulation. These inflammatory mediators could accelerate the progression of systemic organ damage. Previous studies have showed that CRP and IL-6 level was associated with the development of systemic inflammatory response syndrome (SIRS) [29,30]. Furthermore, some studies have confirmed the value of CRP, IL-6 and SIRS on predicting AKI in several clinical settings [31–33]. In brain death organ donors, inflammatory responses mediated by Mitogen-activated protein kinases (MAP-kinases) were considered playing an important role in kidney injury [34]. Therefore, the association between AKI and TBI may similarly be mediated by the activation of systemic inflammation.

Previous studies have illustrated the prognostic role of serum cystatin C in a variety of critically ill patients [35–41]. Non-survivors commonly had higher initial or mean serum cystatin C level than survivors. For patients with neurological diseases, it has been confirmed that level of cystatin C was positively related with severity and adverse outcome of acute ischemic stroke, aneurysmal subarachnoid hemorrhage [42–46]. One study found that concentration of cystatin C in cerebrospinal fluid would increase in TBI patients [47]. Increase of serum cystatin C was also observed in patients suffering acute spinal cord injury [48]. Actually, increased production of cystatin C was an auto neural-protective response to protease hyperactivation in brain tissue and was beneficial for blood-brain barrier integrity and attenuation of early brain injury [47,49–51]. Therefore, we could infer that increased serum cystatin C was partially attributable to brain injury severity and the real value of cystatin C on reflecting renal function may be confounded by the injury severity. However, after adjusting the confounding effects of GCS, an indicator of brain injury severity, in multivariate logistic regression analysis, we found that higher cystatin C was independently associated with occurrence of AKI which indicated that increased cystatin C was an effective and accurate marker of declined renal function in TBI patients.

The mechanism of cystatin C as biomarker of renal function is that it is freely filtered through the glomerulus and completely reabsorbed and catabolized by proximal renal tubular cells. Decreased renal GFR could lead to the accumulation of cystatin C in circulation. Therefore, increased serum cystatin C level could be rationally considered as an indicator of AKI characterized as rapid deterioration of urinary function. Serum creatinine or creatinine clearance was previously acknowledged as an indicator of GFR. However, association between serum creatinine and urinary function of kidney was usually influenced by hemodilution, muscle mass, nutrition, age and sex [12,18,52]. Whereas the fact that serum cystatin C level was less likely to be affected by these factors made it more reliable and effective in detecting early minimal reduction of GFR. In addition, previous studies discovered that life cycle of serum cystatin C was half of that of SCr and increase of cystatin C level above 50% preceded SCr increase by 1.5 days to detect acute renal failure [13,14,53]. Consequently, cystatin C may be a more precise and prompter marker of GFR which could shorten the diagnosis delay in acute clinical settings. Compared with other newly discovered marker of AKI such as TIMP2-IGFBP7 (combination of tissue inhibitor of metalloproteinases 2 and insulin-like growth factor binding protein 7) or NGAL, cystatin C is more readily available. Because physicians could easily obtain the serum cystatin C level from the result of inexpensive blood biochemistry test. In our study, multivariate logistic regression analysis showed that SCr and serum cystatin C both were independent risk factors of AKI in TBI patients. Besides, lymphocyte count was also incorporated in our constructed predictive model. It has been confirmed that various lymphocytes including T cells and B cells both play significant role in initiation, progression and recovery of AKI [54–56]. Neutrophil to lymphocyte ratio (NLR) and platelet to lymphocyte ratio (PLR) were discovered correlated with AKI in many clinical settings [57–60]. RBC transfusion was also confirmed as a latent risk factor for developing AKI in some kinds of patients especially those undergoing cardiac surgery [61–64]. Therefore, incorporating lymphocyte count and RBC transfusion into the predictive nomogram may increase the accuracy and stability. Finally, although use of hypertonic saline was associated with reduced risk of developing AKI in univariate analysis, it was not still statistically significant in multivariate analysis. The correlation between hypertonic saline use and AKI has been studied in many researches without definite conclusions. Some researchers indicated that hypertonic saline could raise serum chloride and sodium concentrations which could cause renal vasoconstriction and consequently decrease renal perfusion [65,66]. However, the correlation between use of hypertonic saline, hyperchloremia and AKI in brain injured patients has not been widely acknowledged [67,68]. One study found prolonged duration of hyperchloremia rather than the duration of hypertonic saline infusion or the highest chloride value was associated with AKI in TBI patients [69]. Another study indicated that neurologically injured patients received infusion of hypertonic saline with a high chloride load were more likely to develop hyperchloremia and AKI [67]. Because no detailed dosage of hypertonic saline was recorded in our study, a further study exploring the relationship between duration of hypertonic saline, serum chloride level and development of AKI is worth to conduct.

There were several limitations in this study. Firstly, this was a single center observational study with relatively small sample size. Further study with larger sample size should be conducted in other medical centers to confirm the predictive value of cystatin C and the constructed nomogram. Secondly, although some confounding factors have been included in this study, some risk factors influencing renal function such as usage of nephrotoxic antibiotics and serum osmolality were not recorded in this study. In addition, history of underlying diseases including hypertension, coronary heart disease and chronic kidney disease were not included into statistical analysis due to the small portion of these diseases. Thirdly, detailed dosage of drugs reducing intracranial pressure including mannitol, fructose glycerol and hypertonic saline was not collected in this study so that we could not accurately evaluate influences of these drugs on renal function. Finally, included TBI patients in this study were mainly diagnosed with moderate to severe TBI and received treatments in NICU. Conclusions of our study should be verified in more generalized TBI patients.

## 5. Conclusions

Serum cystatin C level is independently associated with the development of AKI in TBI patients. Predictive nomogram incorporating cystatin C is effective in evaluating possible occurrence of AKI in TBI patients.

## Ethical approval

This study was approved by the ethics committee of West China hospital and conducted accorded with Declaration of Helsinki. Informed consent form about clinical study of each patients was routinely signed by patients themselves or their legally authorized representatives during their hospitalization.
